# The Pattern of Juvenile Idiopathic Arthritis in a Single Tertiary Center in Saudi Arabia

**DOI:** 10.1155/2016/7802957

**Published:** 2016-02-07

**Authors:** Mohammad H. Al-Hemairi, Shatha M. Albokhari, Mohammed A. Muzaffer

**Affiliations:** ^1^Department of General Pediatrics, Rabigh General Hospital, P.O. Box 51, Rabigh 21911, Saudi Arabia; ^2^Department of Pediatrics, Division of Pediatric Rheumatology, King Abdulaziz University Hospital, P.O. Box. 80218, Jeddah 21589, Saudi Arabia

## Abstract

*Introduction. *Juvenile Idiopathic Arthritis (JIA) is the most common chronic arthritis in children. Our aim is to describe demographic, clinical, and laboratory characteristics and treatment of JIA patients followed up in Pediatric Rheumatology clinic in a tertiary center in Saudi Arabia.* Methods*. Medical records of all patients who are followed up between January 2007 and January 2015 were retrospectively reviewed. Data were collected about demographic, clinical, and laboratory features and treatment.* Results. *Total patients were 82, males were 31 (37.8%), and mean age of JIA onset was 7.1 ± 3.6 yr. Mean follow-up duration was 2.67±1.6 yr. Systemic onset JIA (SoJIA) was the commonest (36.5%), followed by polyarticular in 29.2% and oligoarticular in 28%. Large and small joints are involved in 76 (92%) and 30 (36.6%), respectively. Main extra-articular feature was fever in 34 (41.4%). Uveitis was diagnosed in 7 (8.5%) and in 5 (21.7%) of oligoarticular JIA. Anemia was found in 49 (59.7%), high ESR in 45 (54.8%), and leukocytosis and thrombocytosis in 33 (40.2%). Positive ANA was found in 30 (36.5%) mainly in oligoarticular subtype as 12 (52%) patients (out of 23) had this positive test. 9 patients (10.9%) required NSAIDs only, 6 patients (7.3%) required NSAIDs and intra-articular steroids only, and 19 (23%) required NSAIDs, methotrexate, steroids, and biologics.* Conclusion. *SoJIA is the most common JIA subtype in our study. A population based rather than a single center study will give more details about JIA characteristics in Saudi Arabia

## 1. Introduction

Juvenile Idiopathic Arthritis (JIA) is the most common chronic arthritis in children worldwide. It is a heterogeneous inflammatory disease and defined as arthritis persisting 6 weeks or longer with onset before the age of 16 years with no identifiable etiology.

The term Juvenile Idiopathic Arthritis has replaced the older terms Juvenile Rheumatoid Arthritis (JRA) and Juvenile Chronic Arthritis (JCA) proposed by the International League of Associations for Rheumatology (ILAR) in the late 1990s.

The ILAR classified JIA according to the pattern of the arthritis in the first 6 months after onset of the disease into 8 subtypes known as persistent oligoarticular, extended oligoarticular, polyarticular Rheumatoid Factor- (RF-) negative, polyarticular RF-positive, enthesitis-related arthritis (ERA), psoriatic arthritis, systemic, and undifferentiated arthritis. Each JIA subtype has its own diagnostic criteria (Table 1) [1].

The hallmark of JIA is joint inflammation and the presence of synovitis which causes synovial tissue thickening and accumulation of synovial fluid. This is manifested clinically as joint swelling, morning stiffness, joint pain, tenderness, and functional disability. Arthritis can occur in any joint but large joints are more commonly affected. Joint involvement can be mild and self-limiting and it can be more severe causing joint destruction, severe disability, and loss of joint function.

Extra-articular manifestations in JIA may cause significant morbidity. These include uveitis, fever, skin rash, hepatomegaly, splenomegaly, lymphadenopathy, and serositis. Apart from uveitis, most of these extra-articular features occur in SoJIA.

There are no diagnostic laboratory investigations for JIA but some laboratory findings can help to exclude other causes of arthritis and help in JIA classification.

Aim of JIA management is to control active symptoms and to prevent chronic complications in order to improve quality of life which requires comprehensive multidisciplinary team care including rheumatologists, rehabilitation specialists, occupational therapists, physical therapists, social workers, nurses, podiatrists, dieticians, psychologists or psychiatrists, and orthopedic surgeons.

There is no curative treatment for JIA but early and aggressive management has led to prolonged remission and improved outcome.

Various studies from different parts of the world showed differences in JIA characteristics including incidence, prevalence, age of onset, gender, and frequency of JIA subtypes [2–7].

These relative variabilities may be related to environmental, ethnic, and genetic background.

There is paucity of published studies describing JIA and its characteristics in Saudi Arabia and its neighboring countries. Most of these studies were hospital-based [8–14].

In this present study, we retrospectively reviewed medical records of a cohort of 82 JIA children who are followed up in Pediatric Rheumatology Department at King Abdulaziz University Hospital (KAUH); our objective is to present the main characteristics of JIA in children followed up in our center to know further about the pattern of the disease in Saudi Arabia and to compare these characteristics with those from other parts of the world.

## 2. Patients and Methods

This is an observational retrospective study carried out at Pediatric Rheumatology Department in King Abdulaziz University Hospital (KAUH), a tertiary center, Jeddah, Saudi Arabia.

Our study was approved by local ethical committee of pediatric department and by the University Research Ethics Committee.

All the medical records of the children who were diagnosed to have Juvenile Idiopathic Arthritis (arthritis in one or more joints lasting 2 weeks or more with no identifiable cause in those who are less than 16 years of age) from January 2007 to January 2015 were included.

The diagnosis of JIA was done by a pediatric rheumatologist and JIA classification was according to ILAR classification criteria (Table 1).

Only children who had duration of follow-up of 6 months or more were included in order to know more details about JIA characteristics.

**Figure 1:**
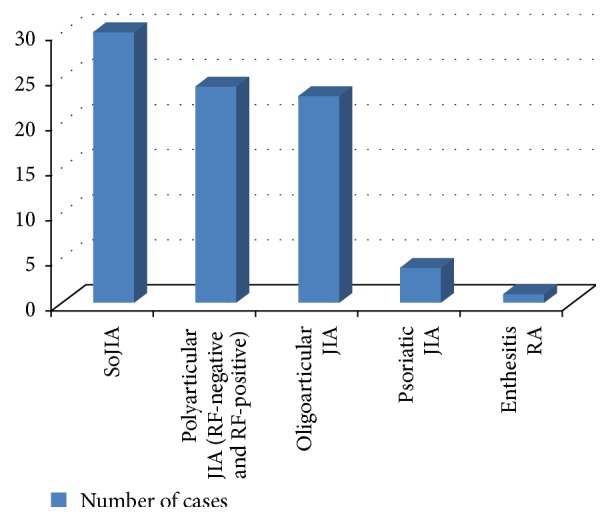
Distribution of JIA subtypes.

Data were collected about number of patients of each JIA subtype, gender, age at disease onset, and duration of follow-up. The percentage of each JIA subtype was calculated and the age of disease onset and duration of follow-up were expressed as the mean ± standard deviation (SD); these data are presented in Table 2 and Figure 1.

Main clinical data were collected about large joints involvement (knee, ankle, elbow, shoulder, and wrist) or small joints involvement and presence of uveitis during the course of the disease. The presence of fever, skin rash, hepatosplenomegaly, and lymphadenopathy at diagnosis was also recorded. These clinical findings were diagnosed by a pediatric rheumatologist while uveitis was diagnosed by ophthalmologist by slit lamp examination. These main clinical data are presented in Table 3.

Laboratory investigations were all done at KAUH laboratories.

Initial data at diagnosis included white blood cells (leukocytosis defined as WBC > 11 × 10^9^/L), hemoglobin level (anemia defined as Hb < 110 g/L), platelets count (thrombocytosis defined as PLT > 450 × 10^9^/L), ESR > 20 mm/hr, elevated CRP > 3 mg/L and positivity of anti-nuclear antibodies (ANA), Rheumatoid Factor (RF), and HLAB27.

ANA was done by ELISA technique and ANA titre of 1:80 or more was considered positive. Positivity of ANA and RF is considered only if two samples were positive in at least three months apart.  Table 4 shows the percentage of these laboratory investigation results in all JIA subtypes.

Review of anti-rheumatic pharmacologic treatments used during the study period included (nonsteroidal anti-inflammatory drugs (NSAIDs), intra-articular corticosteroids (IAC) or systemic corticosteroids, methotrexate (MTX), and biologic agents) was done. Number and percentage of those who were treated with NSAIDs alone, NSAIDs and IAC, NSAIDs, IAC and MTX, NSAIDs and systemic steroids, NSAIDs and MTX, NSAIDs, MTX and systemic steroids, NSAIDs, MTX and biologics, NSAIDs, MTX, and biologics and IAC and those who had joint surgery in each JIA subtype were calculated and presented in Table 5.

Other Disease-Modifying Antirheumatic Drugs (DMARDs) like leflunomide, cyclosporine, and azathioprine were infrequently used and they were not included.

Data statistical analysis was done manually.

## 3. Results

Total patients reviewed in this study were 85; 3 patients were excluded because of insufficient data due to irregular follow-up (2 oligoarticular JIA, 1 polyarticular JIA).

Table 2 shows general characteristics of the patients: systemic onset JIA was the most common JIA subtype 36.5% (30 patients) followed by polyarticular JIA subtype 29.26% (24 patients) and then oligoarticular JIA subtype 28% (23 patients). No unclassified patients JIA were diagnosed.

Of our 82 patients, 51 (62.2%) were females and female gender was predominant in all JIA subtypes except in ERA. Female-to-male ratio was 1.64 : 1 (51/31).

The range of age at onset of symptoms was between 8 months and 14.5 years and the mean age at JIA onset was 7.11 ± 3.65 years.

The duration of follow-up was from 6 months to 8 years; mean was 2.67 ± 1.68 years.

Table 3 shows main clinical features of our patients; large joints were affected in most of patients 92.68% (76/82 patients) while small joints were affected in 36.6% (30/82 patients).

Fever was present in all systemic onset JIA subtype and in 41.46% of the total number (34/82 patients).

Uveitis was diagnosed during the course of the disease in 7 patients (8.53%) and in 5 patients (21.7%) of oligoarticular JIA subtype.

Table 4 shows main laboratory investigations which were done. The most common finding at presentation was anemia in 49/82 patients (59.75%), followed by elevated ESR in 45/82 patients (54.87%) and then elevated CRP and leukocytosis in 33/82 patients (40.24%), while thrombocytosis was found in 31/82 patients (37.8%).

Anemia, leukocytosis, thrombocytosis, elevated ESR, and CRP were present mostly in SoJIA.

Positive ANA was present in 30/82 patients (36.58%) and mostly in oligoarticular JIA 12/23 patients (52.17%) and polyarticular JIA 11/24 patients (45.83%).

Positive Rheumatoid Factor was found only in RF-positive polyarticular JIA.

Table 5 shows the antirheumatic pharmacologic treatment received by our patients during the course of the disease. NSAIDs as the only treating agent were used in 9/82 patients (10.97%) while NSAIDs, methotrexate, systemic steroids, and biologics were received by 19/82 patients (23.17%) at different and varying length of time.

Biologic agents were given to 31/82 patients (37.8%); of these patients, 15 belonged to SoJIA. Biologic agents were mainly in decreasing order of frequency: etanercept (Enbrel), adalimumab (Humira), abatacept (Orencia), rituximab (Mabthera), and tocilizumab (Actemra).

Joint surgery, other than intra-articular corticosteroid injection, was needed in 3 patients only: one case of oligoarticular JIA, RF-negative polyarticular JIA, and RF-positive polyarticular JIA.

## 4. Discussion

This observational study was carried out in Pediatric Department at King Abdulaziz University Hospital, which is a tertiary care hospital in Jeddah, western coast of Saudi Arabia.

We aimed at this study to present the clinical features of children with JIA followed up in our center as knowing the characteristics of this disease in our community is essential to provide a better planning for medical care.

To our best of knowledge, there is only one hospital-based similar study describing the pattern of JIA in Saudi children which was published in 1997 in which arthritis classification was not according to the currently used ILAR classification system for JIA [9].

The prevalence of JIA is variable among different parts of world and different ethnic groups [2, 15]. This variability appears to include the frequency of JIA subtypes.

Unlike most of previous similar studies where oligoarticular JIA was found to be the most common, mainly in Europe, USA, Canada, South America, and Turkey [3, 16–18], the frequency of JIA subtypes in our cohort showed that systemic onset JIA was the most common subtype: 30/82 (36.5%).

This is similar to a previous tertiary hospital-based study done in central province of Saudi Arabia which reviewed clinical features of 115 patients with JRA in which the percentage of SoJIA was found to constitute 44% of Juvenile Rheumatoid Arthritis cases [9]. In a large population-based study in Japan which included 540 children, SoJIA constituted 54% of JIA cases [7].

Our study differs also from two studies from neighboring countries (Kuwait and Oman) in which the most predominant JIA subtype was polyarticular JIA [11, 12].

Polyarticular JIA was also the predominant JIA subtype in some other cohorts from South Africa, Bangladesh, and Pakistan and in a multinational study done by the Paediatric Rheumatology International Trials Organization (PRINTO) which included patients from western and eastern Europe and Latin America [4, 19–21]. ERA was found to be the predominant in other studies [6, 22].

Since our study was done in a tertiary center, we believe that this may affect our results as some cases of mild JIA especially oligoarticular may be treated by general pediatricians or orthopedic doctors and may not be referred to a higher center. We believe also that a single center study may not reflect the correct distribution of JIA subtypes in our country which needs multicenter- or a population-based study.

It is well known that females are generally more commonly affected than males by JIA [23] with variable gender distribution among JIA subtypes [24].

Our present data shows female predominance as 51/82 patients (62.2%) were female. Apart from the only male child with ERA, female number was more in all JIA subtypes.

Few studies showed an overall equal or higher male-to-female ratio [4, 6, 15, 25, 26]; this higher male percentage was possibly related to a higher ERA in one series [3, 25].

The mean age of onset of JIA symptoms in our patients was 7.11 ± 3.65 years (range: 8 months–14.5 years), which is not largely different from most studies [9, 14, 25, 27, 28]. However it is higher than the mean age in a large study which included 3167 patients from Europe and Latin America where mean age of onset of JIA symptoms was 5.8 years [21] but lower than a mean age of 9.5 years recorded in a cohort from Taiwan of 195 patients [22].

It is well known that JIA involves large joints—except hip—more often than small joints; our data showed that 92.68% (76/82) had large joint involvement either alone or associated with small joints.

Fever in SoJIA is considered one of the diagnostic features according to ILAR criteria and it may be the main presenting symptom [9]. As reported in other studies, all our SoJIA patients had fever [8, 29–31].

Among all our JIA children, fever was present in 34/82 (41.46%) patients which is higher than other similar studies [3, 29]. which is probably due to the predominance of SoJIA in our cohort. However a higher percent (67.6%) of fever in JRA was reported in one series [4].

Uveitis is a well-known extra-articular manifestation that affects children with JIA and it occurs more commonly in oligoarticular subtype. It may start before the onset arthritis or later during the course of the disease. Among all our JIA patients 7/82 (8.53%) had uveitis and 5/23 (21.7%) of oligoarticular JIA had developed uveitis. The overall rate is approximately close to what was reported in Spain [32] and Taiwan [22].

However there is significant variability in frequency of uveitis in JIA; in a series of 214 JIA children in India, only 2.2% were found to have uveitis while a rate of 29.65% was reported in another series of 172 patients [33].

Anemia in JIA is commonly caused by iron deficiency or due to chronic inflammation [34].

Anemia was the most common abnormal laboratory investigation in our patients 49/82 (59.75%) and it was recorded in 80% of SoJIA patients. Anemia was considered as hemoglobin level less than 110 g/L which is—according to WHO definition of anemia—a 2SD below the mean for the majority of our patients [35, 36].

The prevalence of anemia in our children may be increased as most of cases were SoJIA in which the anemia is more common than other JIA subtypes.

Furthermore, at presentation, it was not known if this anemia was due to JIA alone or due to other common causes as the frequency of anemia in children in our population was 22.3% and 30% in two local studies [37, 38].

However, although in other studies the cutoff point for low hemoglobin was lower, there were no major differences in the frequency of anemia [3, 10, 14].

Being slightly higher than a local study (115 patients) and studies from UK (572 patients), Turkey (634 patients), and Taiwan (195 patients), the frequency of ANA positivity in our children was 36.58% [3, 9, 22, 39] although higher and more lower frequencies were reported [4, 39].

As expected most ANA positivity cases were in oligoarticular JIA.

The aim of multidisciplinary JIA management is to control active symptoms, achieve remission, prevent joint damage, and preserve joints function to prevent disability as well as maintaining normal growth which may require early and aggressive management [40].

Pharmacologic therapy of JIA has major advances over the last two decades since the introduction of biologic agents which are being increasingly used and these agents may be used as initial therapy [41].

We usually start with NSAIDs for 4 to 6 weeks followed by DMARDs, most commonly methotrexate in case of no adequate response to NSAIDs. IAC are used to relieve joint inflammation and systemic steroids are usually used for a short time with the lowest effective dose and are tapered once we get the desired response. In case of failure of methotrexate, a trial of another DMARD or biologic therapy is introduced.

In our study, all patients were started on NSAIDs either alone or combined with other antirheumatic agents but they were the only used agent in 9/82 patients (11%).

JIA was found to be the most common indication for biologic treatment among pediatric rheumatologic conditions in a national study [42].

As shown in Table 5, 31/82 patients (37.8%) received biologic treatment during their course of the diseases.

A major limitation of our study is being a retrospective record-based in nature and a single center-based with a relatively small sample size. However our study can be a starting point for further future nationwide multicenter-based study.

## 5. Conclusion

In our study, systemic onset JIA is the most common JIA subtype.

Although a population-based rather than a single center study will give more details about JIA characteristics in Saudi Arabia, our study can be the start point to a nationwide multicenter study.

## Figures and Tables

**Table 1:** The ILAR classification of JIA.

Systemic arthritis	Arthritis with, or preceded by, daily fever of at least 2-week duration that is documented to be daily for at least 3 days and accompanied by one or more of the following: (i) evanescent, nonfixed, erythematous rash; (ii) generalized lymph node enlargement; (iii) hepatomegaly and/or splenomegaly; (iv) serositis ^*∗*^Exclusion criteria: A, B, C, and D

Oligoarthritis	Arthritis in 1–4 joints during the first 6 months of disease. Two subtypes: (i) persistent oligoarthritis affects no more than four joints throughout the disease course; (ii) extended oligoarthritis affects a total of more than four joints after the first 6 months of disease ^*∗*^Exclusion criteria: A, B, C, D, and E

Polyarthritis (RF-negative)	Arthritis affecting 5 or more joints during the first 6 months of disease: tests for RF are negative ^*∗*^Exclusion criteria: A, B, C, D, and E

Polyarthritis (RF-positive)	Arthritis in 5 or more joints during the first 6 months of disease: tests for RF are positive ^*∗*^Exclusion criteria: A, B, C, and E

Psoriatic arthritis	Arthritis plus psoriasis or arthritis plus at least two of the following: dactylitis, nail pitting, or onycholysis, psoriasis in a first-degree relative ^*∗*^Exclusion criteria: B, C, D, and E

Enthesitis-related arthritis	Arthritis plus enthesitis or arthritis or enthesitis, plus at least two of the following: presence of or a history of sacroiliac joint tenderness and/or inflammatory lumbosacral pain, presence of HLA-B27 antigen, onset of arthritis in a male over 6 years of age, acute (symptomatic) anterior uveitis, history of AS, ERA, sacroiliitis with IBD, reactive arthritis, or acute anterior uveitis in a first-degree relative ^*∗*^Exclusions criteria: A, D, and E

Undifferentiated arthritis	Arthritis that do not fulfill criteria in any of the above categories or fulfills criteria in two or more of the above categories

^*∗*^ *Exclusion criteria*: A: Psoriasis in the patient or a first-degree relative, B: Arthritis in an HLA-B27-positive male with arthritis onset after 6 years of age, C: Ankylosing spondylitis, enthesitis-related arthritis, sacroiliitis with inflammatory bowel disease, Reiter's syndrome, or acute anterior uveitis in a first-degree relative, D: Presence of IgM rheumatoid factor on at least two occasions for at least 3 months apart, E: Presence of systemic arthritis.

Adapted from © The Journal of Rheumatology Publishing, 2001. All rights reserved. Petty et al. [1].

**Table 2:** General characteristics of JIA patients.

JIA subtype	Number of cases (%)	Gender		Age of onset (yr)		Duration of follow-up (yr)	
		Male (%)	Female (%)	Range	Mean (SD)^*∗*^	Range	Mean (SD)
Oligoarticular	23 (28.04)	10 (43.47)	13 (56.52)	1–12.3	4.78 ± 2.91	0.7–8	2.68 ± 1.86
Polyarticular RF-positive	4 (4.87)	0	4 (100)	10–14.5	12.3 ± 2.78	0.5–5	2.2 ± 1.964
Polyarticular RF-negative	20 (24.39)	6 (30)	14 (70)	2.5–12.4	8.12 ± 3.47	0.6–5.8	2.85 ± 1.376
Systemic	30 (36.5)	13 (43.33)	17 (56.66)	0.7–12.6	7.25 ± 3.47	0.5–6.8	2.37 ± 1.61
Psoriatic	4 (4.87)	1 (25)	3 (75)	4.7–12.2	8.47 ± 3.09	1.3–6.6	3.97 ± 2.17
ERA^*∗*^	1 (1.21)	1 (100)	0	—	—	—	4.8

Total	82 (100)	31 (37.8)	51 (62.2)	0.7–14.5	7.11 ± 3.65	0.5–8	2.67 ± 1.68

^*∗*^ERA: enthesitis-related arthritis.

**Table 3:** Main clinical manifestation of JIA patients.

Clinical manifestations	Oligoarticular number = 23	Polyarticular RF-negative number = 20	Polyarticular RF-positive number = 4	Systemic onset number = 30	Psoriatic number = 4	ERA number = 1	Total number = 82
Large joints involvement	23 (100%)	20 (100%)	3 (75)	27 (90)	2 (50)	1 (100)	**76 (92.68)**
Small joints involvement	4 (17.39)	7 (35)	3 (75)	13 (43.33)	3 (75)	0	**30 (36.6)**
Fever	0	3 (15)	1	30 (100)	0	0	**34 (41.46)**
Uveitis	5 (21.7%)	1 (5)	0	1 (3.33)	0	0	**7 (8.53)**
Skin rash	0	0	0	13 (43.33)	4	0	**17 (20.73)**
Hepatosplenomegaly	0	0	0	11 (36.67)	0	0	**11 (13.41)**
Lymphadenopathy	0	0	0	9 (30)	0	0	**9 (11)**

**Table 4:** Main laboratory investigation.

	Anemia Hb < 110 g/L (%)	Leukocytosis WBC > 11 × 10^9^/L (%)	Elevated PLT 450 × 10^9^/L (%)	Elevated ESR > 20 mm/hr (%)	Elevated CRP > 3 mg/L (%)	Positive ANA (%)	Positive RF	Positive HLA B27 (%)
Systemic onset (number = 30)	24 (80)	21 (70)	21 (70)	24 (85.71)	22 (73.33)	4 (13.33)	0	ND^*∗*^

oligoarticular (number = 23)	9 (39.13)	4 (17.4)	3 (17.64)	7 (30.43)	5 (21.74)	12 (52.17)	0	ND^*∗*^

Polyarticular RF-negative (number = 20)	12 (60)	6 (30)	7 (35)	10 (50)	4 (25)	9 (45)	0	ND^*∗*^

Polyarticular RF-positive (number = 4)	3 (75)	1 (25)	1 (25)	2 (50)	1 (25)	2 (520)	4 (100)	ND^*∗*^

Enthesitis related (number = 1)	0	0	0	1 (100)	1 (100)	0	0	1 (100)

Psoriatic (number = 4)	1 (25)	1 (25)	1 (25)	1 (25)	0	1 (25)	0	ND^*∗*^

Total (number = 82)	49 (59.75)	33 (40.24)	31 (37.8)	45 (54.87)	33 (40.24)	30 (36.58)	4 (4.87)	1 (1.2)

^*∗*^ND: not done.

**Table 5:** Pharmacologic therapy used in JIA patients.

	Oligo. JIA = 23	Poly. RF-neg. = 20	Poly. RF-Pos. = 4	SoJIA = 30	ERA = 1	Psoriatic = 4	Total = 82 (%)
NSAIDs alone	6	2	0	0	1	0	**9 (10.97)**
NSAIDs, AC	4	2	0	0	0	0	**6 (7.31)**
NSAIDs, IAC, MTX	3	0	1	0	0	0	**4 (4.87)**
NSAIDs, steroids	1	0	0	5	0	0	**6 (7.31)**
NSAIDs, MTX	5	0	1	3	0	3	**12 (14.63)**
NSAIDs, MTX, steroids	0	6	0	7	0	1	**14 (17)**
NSAIDs, MTX, biologics	1	5	1	1	0	0	**8 (9.75)**
NSAIDs, MTX, biologics, steroids	2	4	0	13	0	0	**19 (23.17)**
NSAIADs, MTX, biologics, IAC	1	1	1	1	0	0	**4 (4.87)**
Joint surgery	1	1	1	0	0	0	**3 (3.65)**

NSAIDs: Nonsteroidal anti-inflammatory drugs, IAC: intra-articular corticosteroids, and MTX: methotrexate.

## Abbreviations

JIA: Juvenile Idiopathic Arthritis
ILAR: International League of Associations for Rheumatology
RF: Rheumatoid Factor
ANA: Anti-nuclear antibody
SoJIA: Systemic onset Juvenile Idiopathic Arthritis
ERA: Enthesitis-related arthritis
DMARDs: Disease-Modifying Antirheumatic Drugs
NSAIDs: Nonsteroidal anti-inflammatory drugs
MTX: Methotrexate
IAC: Intra-articular corticosteroids.
